# Regional diversity in drug-induced lung diseases among the USA, European Union, and Japan

**DOI:** 10.3389/fmed.2024.1390083

**Published:** 2024-09-24

**Authors:** Jun Sato, Ryo Sadachi, Takafumi Koyama, Yuki Katsuya, Mao Okada, Noboru Yamamoto

**Affiliations:** ^1^Department of Experimental Therapeutics, National Cancer Center Hospital, Tokyo, Japan; ^2^Biostatistics Division, Center for Research Administration and Support, National Cancer Center, Tokyo, Japan

**Keywords:** drug-induced lung disease, FDA adverse event reporting system, ethnic diversity, interstitial lung disease, real-world data

## Abstract

**Background:**

Drug-induced lung disease (DILD) is a considerable and potentially fatal adverse event with poorly understood risk factors. Large-scale, data-driven analyses investigating regional discrepancies in DILD incidence are lacking. The aim of this study was to investigate the potential association among DILD prevalence, regional differences and other factors based on large-scale data base.

**Methods:**

This retrospective observational study analyzed spontaneous adverse event reports from the FDA Adverse Event Reporting System (FAERS) database between January 2010 and December 2020. Regional disparities in DILD incidence were assessed among reports from the United States of America (USA), the European Union (EU), and Japan (JP). Using multivariate logistic regression accounting for age, sex, and reporting years, we calculated the reporting odds ratios (RORs) with 95% confidence intervals. Subgroup analyses were performed for different types of anticancer agents, including tyrosine kinase inhibitors (TKIs), immune checkpoint inhibitors (ICIs), antibody–drug conjugates (ADCs), and cytotoxic agents.

**Results:**

Regional differences in RORs were observed for anticancer drugs in reports from JP and the EU compared with those from the USA (JP, ROR 4.432; EU, ROR 1.291) and for non-anticancer drugs (JP, ROR 3.481; EU, ROR 1.086). Significantly higher RORs were observed for all anticancer drug regimens reported in JP than in the USA (TKIs, ROR 3.274; ICIs, ROR 2.170; ADCs, ROR 2.335; cytotoxic agents, ROR 3.989). The EU reports exhibited higher RORs for TKIs and cytotoxic agents than the USA reports, with no significant differences in ICIs or ADCs (TKIs, ROR 1.679; ICIs, ROR 1.041; ADCs, ROR 1.046; cytotoxic agents, ROR 1.418).

**Conclusion:**

The prevalence of DILD in JP, the EU, and the USA differed. These findings have important implications in evaluating the safety profiles of drugs and patient safety in drug development and clinical practice. This study is the first to identify regional differences in DILDs using a large global database.

## 1 Introduction

Drug-induced lung disease (DILD) is an adverse event (AE) with a fatality rate of up to 40% (1–3). Anticancer drugs are the most common cause of DILDs, and their prevalence has increased with the use of recently developed anticancer drugs (3). Specifically, the occurrence rate of DILDs in patients administered with tyrosine kinase inhibitors (TKIs) and immune checkpoint inhibitors (ICIs) is 5–20%, which is higher than the approximately 2% rate in patients administered with classical cytotoxic agents (2, 4). The mortality rate of DILDs associated with anticancer drugs, particularly TKIs and ICIs, is extremely high, with mortality rates of 35–40% and 15–20%, respectively (2, 5). Although DILDs have a considerable clinical impact, their associated predictive factors have not been elucidated.

Regional differences in DILD incidence have been noted in several clinical trials, with relatively high incidences reported in Asia (4). The incidence of DILDs after treatment with molecularly targeted drugs [e.g., epidermal growth factor receptor (EGFR) TKIs] in Asia is 10 times higher than that in non-Asian regions. The mortality rate of DILDs associated with molecularly targeted drugs is higher than that of DILDs associated with drugs with other mechanisms of action (1). For antibody–drug conjugates (ADCs) and ICIs, the incidence of DILDs is relatively high in Asian (Japanese) patients. In clinical studies of ICIs in Asia (Japan), regional differences in DILD prevalence have been observed, with a reported rate of 15–20% (4, 6–8).

Large-scale database analyses investigating the regional differences in DILDs have not yet been reported. The Food and Drug Administration Adverse Event Reporting System (FAERS) is the largest database of spontaneous reports on drug-related AEs worldwide. However, no previous reports have employed these types of large-scale databases to examine ethnic differences in DILD development. This study aimed to investigate the potential associations among DILD prevalence, regional differences, and other factors by using FAERS data.

## 2 Materials and methods

### 2.1 Study design and data source

This retrospective observational study was conducted using data from the FAERS database. FAERS data were downloaded through the USA Food and Drug Administration (FDA) website,^1^ and DILD reports issued between January 2010 and December 2020 were identified. As of January 2023, more than 14 million reports have been collected and analyzed by downloading raw data. AEs suspected to be associated with DILD in the FAERS were reviewed separately. The following information was collected: patient characteristics (date of AE, date of report to the FDA, age at AE, sex, weight, reporter, reporting country, and country of occurrence), drug information (suspected drug identification, drug name, route of administration, dose, administration date, treatment start date, and treatment end date), patient outcomes (death and hospitalization), and source of information (foreign, clinical trial, and consumer). The FAERS is a publicly accessible, anonymous database. The requirement for informed consent and approval from the institutional review board was waived.

### 2.2 Data collection and definition

We investigated AEs reported between January 2011 and December 2020 in patients aged 20–100 years at the time of AE occurrence. In the FAERS, the Medical Dictionary of Regulatory Activities organ system classification and preferred term level were used to describe suspected AEs.

The investigators (respiratory physicians) reviewed all reported AEs and defined DILD items that could be clinically determined as DILDs. A comprehensive inventory of DILD items was compiled (Supplementary Table S1).

All reported drugs were reviewed by the investigators and classified as either anticancer or non-anticancer. Anticancer drugs were further classified into TKIs, ICIs, ADCs, antibodies, cytotoxins, hormones, and others according to their mechanisms of action (Supplementary Table S2). The causal relationship between a combined regimen (drugs with different modes of action) remains unclear, and only monotherapeutic anticancer drugs or drugs with the same known mechanisms of action were included in the analyses.

Regional differences in DILD incidence were examined using data from the USA, the European Union (EU), and Japan (JP) reports. The names of the reporting countries were collected from the FAERS data, and the EU included member countries as of August 2022.^2^ Members of the EU were identified and listed by country codes, and the program automatically identified matching records.

### 2.3 Statistical analysis

Patient characteristics in each region were collected as follows. The median and the interquartile range were calculated for continuous patient characteristics. For categorical patient characteristics, frequencies were tabulated.

The risk of DILDs in the EU and JP compared with that in the USA was assessed using the reporting odds ratio (ROR), defined as the ratio of the odds in reports from the JP or the EU to that in the USA, and the odds were defined as the ratio of the presence of DILD to the absence of DILDs in each region. An ROR close to one indicated no difference in the frequency of DILDs between the EU (or JP) and the USA reports. When the ROR was >1, the risk of DILDs in the EU (or JP) reports was higher than that in the USA reports.

A logistic regression model was used to calculate the DILDs and 95% confidence intervals (CIs) for the EU and JP reports relative to those for the USA reports. Multivariate analysis was performed using a logistic regression model to adjust for confounding variables, including sex (male/female), age (10-year range), and year of occurrence (2010–2014 or 2015–2020). To assess the risk of DILDs by drug type, we performed the same analysis for drug types (i.e., non-anticancer and anticancer drugs) with a reasonable prevalence in single agents or agents with the same mechanism of action (TKIs, ICIs, ADCs, and cytotoxic agents) in subgroup analyses. All analyses were performed using SAS version 9.4.

## 3 Results

### 3.1 Patient demographics

**Figure 1:**
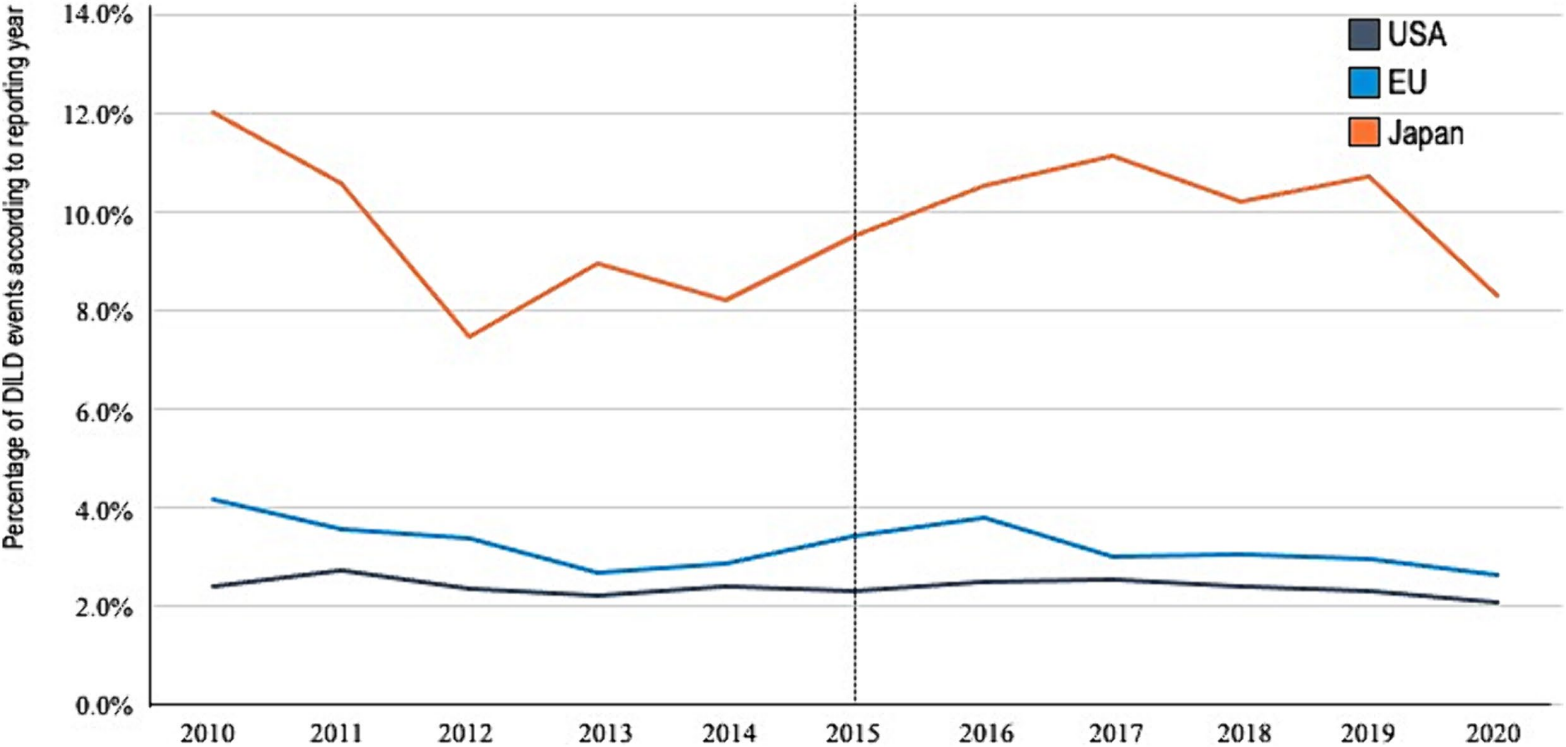
Reporting percentage by year. DILDs, drug-induced lung disease; US, United States; EU, European Union; JP, Japan.

We identified patients with AEs and DILDs in reports from the USA [3,057,102 and 73,137 (2.39%), respectively], EU [934,576 and 29,533 (3.16%), respectively], and JP [228,064 and 22,463 (9.84%), respectively]. Sex-reporting bias was also observed. The reports indicated relatively older age in JP, fewer fatalities and more consumer reports in the USA, and more patients with onset within 90 days in JP. No other significant differences in characteristics were found among the reports from the three regions (Table 1). No significant difference was noted in the reporting proportion (DILD events per total AEs) based on the reported years (Figure 1).

**Table 1:** Country-wise characteristics of patients with DILDs in the FAERS (data available).

	USA	EU	JP
Total reports of AE	10,417,014	3,363,540	743,229
Total reports of DILD	73,137	29,533	22,463
Sex			
Male	29,674	15,513	14,244
Female	43,108	13,804	8,062
Unknown/missing	355	216	157
Age (years)			
Median (IQR)	65 (56–74)	67 (56–75)	71 (63–78)
Reporting year			
2010–2014	27,222	9,518	8,745
2015–2020	45,915	20,015	13,718
Outcome			
Fatal	4,439	3,663	3,491
Other/Missing	68,698	25,870	18,972
Type of reporter			
Physician	15,585	14,710	15,081
Consumer	30,520	4,409	3,018
Pharmacist	7,690	1,366	1,735
Other/Missing	15,585	14,710	15,081
Time to onset (days)			
<90	15,555	10,878	12,600
90–365	9,909	4,582	3,620
≥ 365	12,849	3,961	1,775
Drugs			
Non-anticancer drug	48,750	15,354	9,711
Anticancer drug	24,387	14,179	12,752
TKIs	4,489	2,047	2,160
ICIs	1,144	1,595	3,177
ADCs	88	78	64
Cytotoxics	3,359	3,778	2,269

AE, adverse event; DILDs, drug-induced lung diseases; FAERS, Food and Drug Administration Adverse Event Reporting System; US, United States; EU, Europe; JP, Japan; TKIs, tyrosine kinase inhibitors; ICIs, immune checkpoint inhibitors; ADCs, antibody–drug conjugates; cytotoxics, cytotoxic drugs; IQR, interquartile range.

### 3.2 Reported regions and DILDs

**Figure 2:**
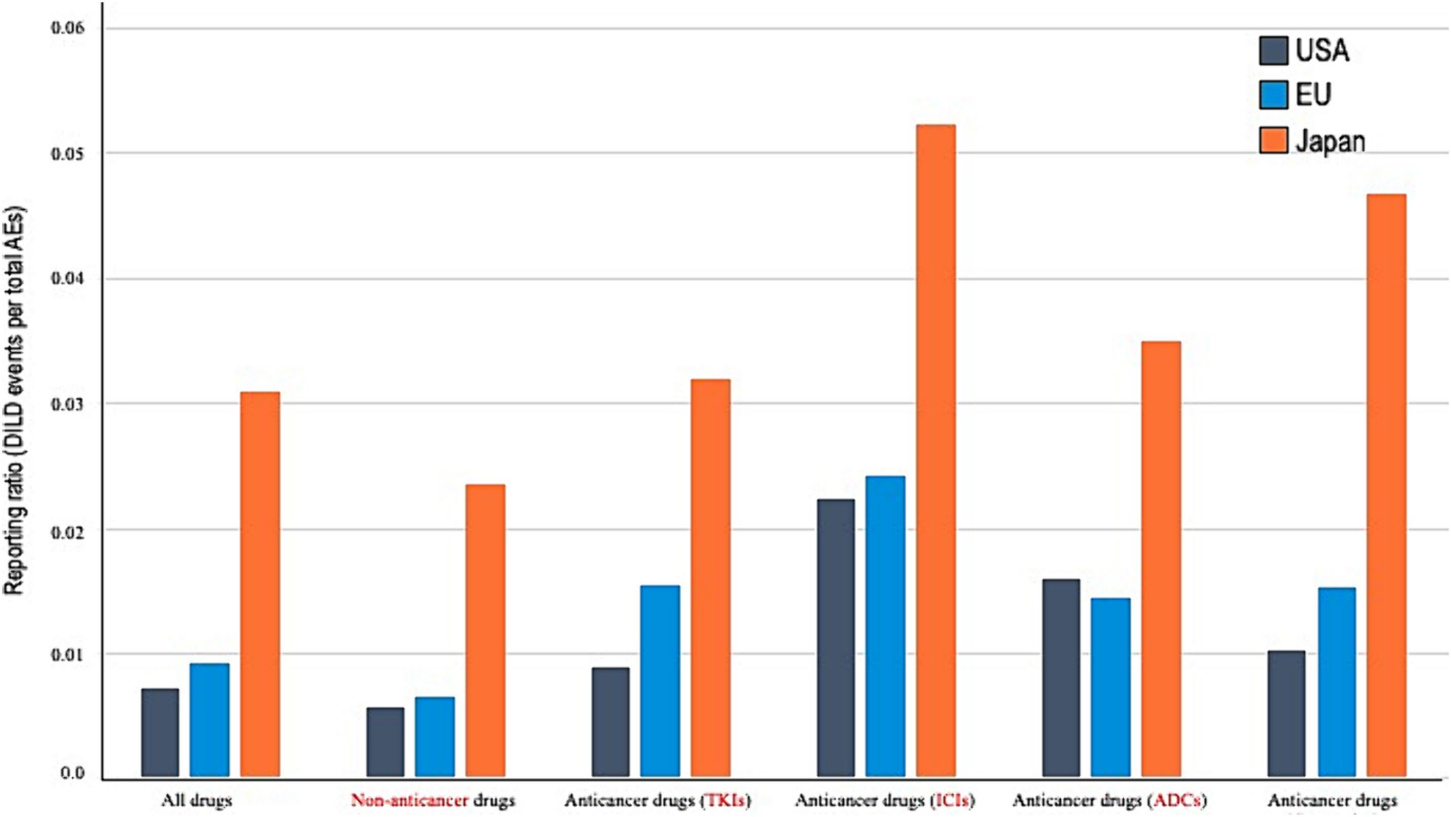
Reporting proportion according to the drug types. AE, adverse event; DILDs, drug-induced lung disease; US, United States; EU, European Union; JP, Japan; TKIs, tyrosine kinase inhibitors; ICIs, immune checkpoint inhibitors; ADCs, antibody–drug conjugates; cytotoxics, cytotoxic agents.

Univariate analysis results (Table 2) indicated that the incidence of DILDs was relatively low in females and increased with age. As shown in Figure 2 and Table 3, multivariate analysis revealed a higher reporting proportion and RORs of non-anticancer drugs in JP than in the USA. By contrast, no difference in DILD incidence rates associated with non-anticancer drugs was found between reports from the EU and USA. Significant differences in RORs were also observed across anticancer drugs in reports from JP and the EU compared with those from the USA (JP, ROR 4.432 [95% CI, 4.366–4.499]; EU, ROR 1.291 [95% CI, 1.274–1.308]). The fatality rates associated with DILDs in the USA, EU, and JP reports were 6.1, 12.4, and 15.5%, respectively, with a significantly higher proportion of DILDs in the EU and JP reports than in the USA reports.

**Table 2:** Univariate analysis of DILDs reported in the FAERS database.

	No. of DILD	Univariable analysis	
		Reporting odds ratio (95% CI)	*P*
Sex			
Male	61,522	1	
Female	67,039	0.616 (0.609–0.623)	<0.0001
Age group			
20–29	2,512	1	
30–39	4,741	1.309 (1.247–1.374)	<0.0001
40–49	9,973	1.907 (1.825–1.992)	<0.0001
50–59	21,591	2.590 (2.485–2.699)	0.0004
60–69	35,683	3.733 (3.585–3.887)	<0.0001
70–79	36,388	4.882 (4.688–5.084)	<0.0001
80–89	15,799	4.845 (4.645–5.054)	<0.0001
90–99	1,874	4.392 (4.136–4.664)	
Years			
2010–2014	46,747	1	
2015–2020	81,814	1.018 (1.007–1.030)	0.0019
Region			
USA	74,548	1	
EU	31,009	1.291 (1.274–1.308)	<0.0001
JP	23,004	4.432 (4.366–4.499)	<0.0001

DILDs, drug-induced lung diseases; FAERS, Food and Drug Administration Adverse Event Reporting System; USA, United States of America; EU, Europe; JP, Japan; CI, confidence interval.

**Table 3:** Regional differences of DILDs reported in the FAERS database.

	No. of DILDs	Multivariate analysis	
		Reporting odds ratio (95% CI)	*P*
All drugs			
USA	74,548	1	
EU	31,009	1.218 (1.202–1.234)	<0.0001
JP	23,004	3.608 (3.553–3.664)	<0.0001
Non-anticancer drug			
USA	49,707	1	
EU	16,103	1.086 (1.066–1.106)	<0.0001
JP	9,900	3.481 (3.404–3.560)	<0.0001
Anticancer drug (TKIs)			
USA	4,630	1	
EU	2,176	1.679 (1.594–1.769)	<0.0001
JP	2,232	3.274 (3.105–3.451)	<0.0001
Anticancer drug (ICIs)			
USA	1,221	1	
EU	1,685	1.041 (0.965–1.123)	0.2960
JP	3,308	2.170 (2.025–2.325)	<0.0001
Anticancer drug (ADCs)			
USA	93	1	
EU	78	1.046 (0.763–1.434)	0.7804
JP	65	2.335 (1.666–3.272)	<0.0001
Anticancer drug (Cytotoxics)			
USA	3,511	1	
EU	4,043	1.418 (1.354–1.485)	<0.0001
JP	2,329	3.989 (3.776–4.215)	<0.0001

DILDs, drug-induced lung diseases; FAERS, Food and Drug Administration Adverse Event Reporting System; US, United States; EU, Europe; JP, Japan; TKIs, tyrosine kinase inhibitors; ICIs, immune checkpoint inhibitors; ADCs, antibody–drug conjugates; cytotoxics, cytotoxic agents; CI, confidence interval.

### 3.3 Subgroup analysis

The results of multivariate analysis for each single-agent regimen of anticancer drugs showed that the RORs in reports from JP were significantly higher than those in reports from the USA (TKIs, ROR 3.274 [95% CI, 3.105–3.451]; ICIs, ROR 2.170 [95% CI, 2.025–2.325]; ADCs, ROR 2.335 [95% CI, 1.666–3.272]; and cytotoxic agents, ROR 3.989 [95% CI, 3.776–4.215]). Reports from the EU had higher RORs than those from the USA for TKIs and cytotoxic agents; no statistically significant differences were noted between the RORs of ICIs and ADCs (TKIs, ROR 1.679 [95% CI, 1.594–1.769]; ICIs, ROR 1.041 [95% CI, 0.965–1.123]; ADCs, ROR 1.046 [95% CI, 0.763–1.434]; and cytotoxic agents, ROR 1.418 [95% CI, 1.354–1.485]).

## 4 Discussion

This study is the first to identify regional differences in DILDs using a large, global database. This analysis revealed that the prevalence of DILDs was significantly higher in JP than in the USA for all anticancer and non-anticancer drugs. This study confirmed the previously reported higher prevalence of DILDs in clinical trials due to TKIs and ADCs as well as the previously unreported higher prevalence of DILDs due to ICIs in JP than in Western countries (4, 6–8). The EU had higher RORs than the US for TKIs and cytotoxic agents, and no significant differences in RORs were observed between ICIs and ADCs.

Previous clinical trials have shown that Asians, specifically Japanese, are susceptible to DILDs after treatment with certain drugs, and the incidence of DILDs caused by gefitinib, an EGFR-TKI, is relatively high in the Asian population (9). Similar trends have been reported for ADCs and TKIs, with Asians having a relatively high risk of developing DILDs (3). In the present study, a discernible discrepancy in the incidence rates of DILDs between reports from JP and the USA was observed. This disparity has been observed in the contexts of TKIs and ADCs. In terms of ICIs, a few previous studies have reported ethnic discrepancies in DILD prevalence that, as delineated in previous reports, ranges between 14.5 and 19.0% (7, 10, 11). This study elucidated a notable distinction in the incidence rates of DILDs across various drug types, including ICIs, when comparing reports from JP and the USA, using a large-scale database. A similar trend surfaced when comparing reports from JP, China, South Korea, and ASEAN countries with those from the EU and the USA (Supplementary Table S3). These findings implicate inherent factors, such as genomic variances and comorbid conditions, as potential drivers of discrepancies in DILD prevalence after treatment with anticancer drugs, including ICIs, between Asian and non-Asian populations.

This study investigated the differences in reporting propensity from JP, the EU, and the USA by comparing the reports from the three regions to ensure data robustness. The data among the three regions were possibly influenced by biases in reporting, healthcare access, language, and DILD severity. These biases were considered when interpreting the results of this study. We investigated reporter bias by examining the differences in reporting between the EU and the USA, considering reporting bias from regions other than the USA. The RORs of ICIs and ADCs showed only minor differences between the EU and USA, whereas clear differences were observed between the USA and JP. The high proportion of healthcare professionals as reporters in both the EU and Japan contribute to the higher number of severe cases being reported from these regions (Table 1). Regional differences in mortality from DILDs were notably higher in JP and the EU than in the USA. This result indicates that reporting relatively mild cases of DILDs does not result in a reporting bias that would increase the prevalence in JP and the EU. Moreover, significant DILDs that needed to be reported were recorded regardless of the region and country, including outside the USA. These findings eliminated reporting bias and strongly suggested ethnic differences in DILD incidence resulting from several agents.

The mechanisms by which anticancer drugs induce DILDs must be elucidated for the prevention and treatment of this AE. The results of the present study showed that the prevalence of DILD differed with each drug, indicating that the mechanism underlying DILD development differs for each drug. Two main mechanisms of DILD development have been proposed: direct drug-induced damage and immunological mechanisms. Several hypotheses on the mechanism of direct injury state that drugs induce the release of cytotoxic reactive oxygen species in the lungs, leading to the degeneration of alveolar cells and pulmonary macrophages owing to increased phospholipid accumulation in alveolar cells (1, 2, 12–15). In terms of immunological mechanisms, direct haptenic modification of tissue-resident proteins by immune cells and deposition of antigen–antibody complexes have been proposed (8, 12, 16). Few studies reported on the mechanism of DILD development using tissue samples from affected patients. Further research on each mode of action should be conducted using blood and tissue samples and various omics analyses to identify predictive biomarkers for DILD development, especially in Asians, where DILD is more common. Our team has initiated a study analyzing the immunological profile and genetic background of DILD tissue samples.

In conclusion, the prevalence of DILD was higher in JP than in the USA and EU across all anticancer drug regimens. These findings underscore the importance of considering differences in the prevalence of DILD in JP, the EU, and the USA when evaluating the safety profiles of drug development and patient safety in clinical practice.

## Data Availability

The original contributions presented in the study are included in the article/Supplementary material, further inquiries can be directed to the corresponding author.

## Footnotes

^1^https://fis.fda.gov/extensions/FPD-QDE-FAERS/FPD-QDE-FAERS.html

^2^https://www.eeas.europa.eu/_en?s=169
