# Supplementary material for: Regional diversity in drug-induced lung diseases among the USA, European Union, and Japan
Source: Front Med (Lausanne). 2024 Sep 24;11:1390083. doi: 10.3389/fmed.2024.1390083 (PMC11458412; doi:10.3389/fmed.2024.1390083)
Supplement: Supplementary file 1 [file Data_Sheet_1.docx]

Supplementary Material

# Supplementary Tables

**Table S1.** List of adverse events identified as DILDs.

| Acute interstitial pneumonitis  Autoimmune lung disease |
| --- |
| Eosinophilic bronchitis  Eosinophilic pneumonia |
| Eosinophilic pneumonia acute |
| Idiopathic interstitial pneumonia  Idiopathic pulmonary fibrosis |
| Immune-mediated pneumonitis |
| Interstitial lung disease |
| Organizing pneumonia  Pneumonitis |
| Pulmonary fibrosis |

Abbreviations: DILDs, drug-induced lung disease.

**Table S2.** Classification of anticancer drugs based on their mechanisms of action.

| Mode of action | Drug name |
| --- | --- |
| TKI | Gefitinib, Erlotinib, Afatinib, Dacomitinib, Osimertinib, Crizotinib, Ceritinib, Alectinib, Lorlatinib, Brigatinib, Dabrafenib, Trametinib, Encorafenib, Binimetinib, Vemurafenib, Vemurafenib, Selpercatinib, Entrectinib, Tepotinib, Capmatinib, Vandetanib, Sotorasib, Imatinib, Imatinib, Lenvatinib, Sunitinib, Sorafenib, Sorafenib, Axitinib, Cabozantinib, Abemaciclib, Palbociclib, Niraparib, Olaparib, Regorafenib, Pazopanib, Temsirolimus, Everolimus, Nilotinib, Nilotinib, lapatinib, Lapatinib, Dasatinib, Bosutinib, Ponatinib, Ruxolitinib, Ibrutinib, Gilteritinib, Tivozanib, Umbralisib, Pralsetinib, Ripretinib, Pemigatinib, Tucatinib, Tazemetostat, Zanubrutinib, Alpelisib, Erdafitinib, Larotrectinib, Talazoparib, Duvelisib, Ivosidenib, Acalabrutinib, Copanlisib, Enasidenib, Neratinib, Midostaurin, Ribociclib, Rucaparib, Venetoclax, Cobimetinib, Idelalisib |
| ICI | Nivolumab, Pembrolizumab, Ipilimuma, Atezolizumab, Durvalumab, Avelumab, Dostarlimab, Cemiplimab |
| ADC | Trastuzumab Emtansine, Ado-trastuzumab emtansine, Trastuzumab Deruxtecan, Enfortumab Vedotin, Brentuximab Vedotin, Ibritumomab Tiuxetan, Gemtuzumab Ozogamicin, Inotuzumab Ozogamicin, Loncastuximab tesirine, Belantamab mafodotin, Sacituzumab govitecan, Polatuzumab vedotin, Moxetumomab pasudotox |
| Cytotoxic | Irinotecan Hydrochloride Hydrate, Trabectedin, Streptozocin, Cabazitaxel Acetonate, Trifluridine Tipiracil Hydrochloride, Eribulin Mesilate, Pemetrexed sodium hydrate, Pemetrexed disodium heptahydrate, Temozolomide, Oxaliplatin, Ifosfamide, Cyclophosphamide Hydrate, Melphalan, Melphalan flufenamide hydrochloride, Bendamustine hydrochloride hydrate, Busulfan, Ranimustine, Nimustine hydrochloride, Carboplatin, Carmustine, Dacarbazine, Procarbazine hydrochloride, Bleomycin, Hydrochloride, Mitomycin C, Actinomycin D, Cisplatin, Nedaplatin, Fluorouracil, Tegafur, Doxifluridine, Capecitabine, Doxorubicin hydrochloride, Cytarabine, Cytarabine Ocfosphate hydrate, Enocitabine, Gemcitabine hydrochloride, Nelarabine, Azacitidine, Mercaptopurine hydrate, Fludarabine phosphate, Cladribine, Methotrexate, Pralatrexate, Hydroxycarbamide, Irinotecan hydrochloride hydrate, Epirubicin Hydrochloride, Daunorubicin hydrochloride, Idarubicin hydrochloride, Pirarubicin, Amrubicin hydrochloride, Aclarubicin hydrochloride, Mitoxantrone hydrochloride, Etoposide, Sobuzoxane, Vincristine Sulfate, Vinblastine Sulfate, Vindesine Sulfate, Paclitaxel, Lurbinectedin |
| Antibody | Pertuzumab, Bevacizumab, Ramucirumab, Panitumumab, Necitumumab, Aflibercept, Aflibercept Beta, Ziv-Aflibercept, Cetuximab, Rituximab, Obinutuzumab, Ofatumumab, Daratumumab, Alemtuzumab, Trastuzumab, Elotuzumab, Mogamulizumab, Blinatumomab, Amivantamab, Mogamulizumab, Margetuximab, Tafasitamab, Isatuximab, Tagraxofusp, Olaratumab, Elotuzumab, Necitumumab, Dinutuximab |
| Hormone | Tamoxifen citrate, Toremifene citrate, Fulvestrant, Anastrozole, Letrozole, Exemestane, Medroxyprogesterone acetate, Goserelin acetate, Leuprorelin acetate, Degarelix acetate, Flutamide, Bicalutamide, Chlormadinone acetate, Enzalutamide, Abiraterone Acetate, Ethinylestradiol, Estramustine phosphate sodium hydrate, Octreotide acetate, Lanreotide acetate, Mitotane, Drospirenone and estetrol, Relugolix, Darolutamide, Apalutamide |
| Others | thalidomide, Lenalidomide hydrate, Pomalidomide, Interferon, Teceleukin, Tretinoin, Tamibarotene, Arsenic trioxide, L-Asparaginase, Bortezomib, Vorinostat, Panobinostat Lactate, Romidepsin, Ixazomib citrate, Carfilzomib, Forodesin, Bexarotene, Radium (223Ra) chloride, Decitabine and cedazuridine, Pexidartinib hydrochloride, Selinexor, Calaspargase pegol, Glasdegib maleate, Emapalumab, Lutetium (177Lu) oxodotreotide, Sonidegib, Belinostat, Omacetaxine mepesuccinate, Asparaginase Erwinia chrysanthemi, Ruxolitinib, Plerixafor |

Abbreviations: TKI, tyrosine kinase inhibitor; ICI, immune checkpoint inhibitor; ADC, antibody-drug conjugate; cytotoxic, cytotoxic agent.

**Table S3.** Subgroup analysis in FAERS (Asia vs. the US vs. the EU).

|  | N of ILD/Reported | Univariable analysis | | Multivariable analysis | |
| --- | --- | --- | --- | --- | --- |
|  |  | Odds ratio  (95% CI) | *P* | Odds ratio  (95% CI) | *P* |
| Sex |  |  |  |  |  |
| Male | 63,821/5,377,438 | 1 |  | 1 |  |
| Female | 68,440/9,372,887 | 0.612  (0.606-0.619) | < 0.0001 | 0.734  (0.725–0.742) | < 0.0001 |
| Age group |  |  |  |  |  |
| 20–29 | 2,600/911,151 | 1 |  | 1 |  |
| 30–39 | 4,935/1,316,718 | 1.315  (1.254-1.379) | < 0.0001 | 1.287  (1.227–1.350) | < 0.0001 |
| 40–49 | 10,320/1,905,306 | 1.903  (1.823-1.987) | < 0.0001 | 1.841  (1.763–1.922) | < 0.0001 |
| 50–59 | 22,299/3,039,281 | 2.583  (2.480-2.690) | < 0.0001 | 2.492  (2.392–2.595) | < 0.0001 |
| 60–69 | 36,805/3,496,401 | 3.718  (3.572-3.869) | < 0.0001 | 3.385  (3.253–3.523) | < 0.0001 |
| 70–79 | 37,271/2,730,488 | 4.836  (4.647-5.032) | < 0.0001 | 4.418  (3.985–4.317) | < 0.0001 |
| 80–89 | 16,112/1,194,908 | 4.776  (4.582-4.979) | < 0.0001 | 4.021  (3.857–4.192) | < 0.0001 |
| 90–99 | 1,919/156,072 | 4.350  (4.100-4.616) | < 0.0001 | 3.665  (3.454–3.889) | < 0.0001 |
| Years |  |  |  |  |  |
| 2010–2014 | 47,702/5,405,044 | 1 |  | 1 |  |
| 2015–2020 | 84,559/9,345,281 | 1.025  (1.014-1.037) | < 0.0001 | 0.982  (0.971–0.993) | 0.0020 |
| Country |  |  |  |  |  |
| USA | 74,548/10,353,889 | 1 |  | 1 |  |
| EU | 31,009/3,363,540 | 1.291  (1.274-1.308) | < 0.0001 | 1.217  (1.201–1.233) | < 0.0001 |
| Asia* | 26,704/969,771 | 3.930  (3.875-3.986) | < 0.0001 | 3.301  (3.245–3.349) | < 0.0001 |

*Japan, China, Korea, and the ASEAN

Reporting Odds ratios (ORs) in the US, EU, and Asia by drug type

**TKIs**

|  | *N* of ILD/Reported | Univariable analysis | | Multivariable analysis | |
| --- | --- | --- | --- | --- | --- |
|  |  | Odds ratio  (95% CI) | *P* | Odds ratio  (95% CI) | *P* |
| Country |  |  |  |  |  |
| USA | 4,630/522,537 | 1 |  | 1 |  |
| EU | 2,176/140,542 | 1.759  (1.671–1.852) | < 0.0001 | 1.682  (1.597–1.772) | < 0.0001 |
| Asia | 2,609/92,050 | 3.263  (3.108–3.425) | < 0.0001 | 2.958  (2.814–3.109) | < 0.0001 |

**ICIs**

|  | *N* of ILD/Reported | Univariable analysis | | Multivariable analysis | |
| --- | --- | --- | --- | --- | --- |
|  |  | Odds ratio  (95% CI) | *P* | Odds ratio  (95% CI) | *P* |
| Country |  |  |  |  |  |
| USA | 1221/54,400 | 1 |  | 1 |  |
| EU | 1685/69,482 | 1.082  (1.005–1.166) | 0.0372 | 1.042  (0.966–1.124) | 0.2855 |
| Asia | 3611/68,229 | 2.434  (2.279–2.600) | < 0.0001 | 2.212  (2.066–2.369) | < 0.0001 |

**ADCs**

|  | *N* of ILD/Reported | Univariable analysis | | Multivariable analysis | |
| --- | --- | --- | --- | --- | --- |
|  |  | Odds ratio  (95% CI) | *P* | Odds ratio  (95% CI) | *P* |
| Country |  |  |  |  |  |
| USA | 93/5795 | 1 |  | 1 |  |
| EU | 78/5364 | 0.905  (0.668–1.225) | 0.5175 | 1.045  (0.762–1.431) | 0.7862 |
| Asia | 75/2124 | 2.245  (1.649–3.056) | < 0.0001 | 2.404  (1.737–3.325) | < 0.0001 |

**Cytotoxic**

|  | *N* of ILD/Reported | Univariable analysis | | Multivariable analysis | |
| --- | --- | --- | --- | --- | --- |
|  |  | Odds ratio  (95% CI) | *P* | Odds ratio  (95% CI) | *P* |
| Country |  |  |  |  |  |
| USA | 3511/341,737 | 1 |  | 1 |  |
| EU | 4043/262,826 | 1.505  (1.438–1.575) | < 0.0001 | 1.415  (1.351–1.482) | < 0.0001 |
| Asia | 2742/66,814 | 4.123  (3.919–4.337) | < 0.0001 | 3.628  (3.445–3.821) | < 0.0001 |

**Antibody**

|  | *N* of ILD/Reported | Univariable analysis | | Multivariable analysis | |
| --- | --- | --- | --- | --- | --- |
|  |  | Odds ratio  (95% CI) | *P* | Odds ratio  (95% CI) | *P* |
| Country |  |  |  |  |  |
| USA | 765/86,616 | 1 |  | 1 |  |
| EU | 1038/58,308 | 2.034  (1.852–2.234) | < 0.0001 | 1.845  (1.677–2.029) | < 0.0001 |
| Asia | 700/22,186 | 3.656  (3.296–4.055) | < 0.0001 | 3.079  (2.769–3.423) | < 0.0001 |

**Hormone**

|  | *N* of ILD/Reported | Univariable analysis | | Multivariable analysis | |
| --- | --- | --- | --- | --- | --- |
|  |  | Odds ratio  (95% CI) | *P* | Odds ratio  (95% CI) | *P* |
| Country |  |  |  |  |  |
| USA | 811/172,908 | 1 |  | 1 |  |
| EU | 343/55,327 | 1.324  (1.166–1.502) | < 0.0001 | 1.464  (1.287–1.667) | < 0.0001 |
| Asia | 911/233,702 | 8.483  (7.710–9.335) | < 0.0001 | 8.299  (7.528–9.149) | < 0.0001 |

**Others**

|  | *N* of ILD/Reported | Univariable analysis | | Multivariable analysis | |
| --- | --- | --- | --- | --- | --- |
|  |  | Odds ratio  (95% CI) | *P* | Odds ratio  (95% CI) | *P* |
| Country |  |  |  |  |  |
| USA | 10,509/346,389 | 1 |  | 1 |  |
| EU | 1143/58,739 | 0.634  (0.596–0.675) | < 0.0001 | 0.687  (0.645-0.731) | < 0.0001 |
| Asia | 1021/36,337 | 0.924  (0.866–0.986) | 0.0175 | 0.929  (0.870-0.992) | 0.0269 |

**Non-anticancer drugs**

|  | *N* of ILD/Reported | Univariable analysis | | Multivariable analysis | |
| --- | --- | --- | --- | --- | --- |
|  |  | Odds ratio  (95% CI) | *P* | Odds ratio  (95% CI) | *P* |
| Country |  |  |  |  |  |
| USA | 49,707/8,661,630 | 1 |  | 1 |  |
| EU | 16,103/2,451,911 | 1.145  (1.125–1.166) | < 0.0001 | 1.085  (1.065–1.104) | < 0.0001 |
| Asia | 11,524/573,059 | 3.556  (3.485–3.630) | < 0.0001 | 3.049  (2.986–3.113) | < 0.0001 |
